# Examining the impact of distance as a contextual cue in evaluative conditioning

**DOI:** 10.1371/journal.pone.0204855

**Published:** 2018-10-04

**Authors:** Sean Hughes, Simone Mattavelli, Jan De Houwer

**Affiliations:** Department of Experimental Clinical and Health Psychology, Ghent University, Ghent, Belgium; Universidad de Granada, SPAIN

## Abstract

According to a symbolic perspective on EC, pairings constitute a relational contextual cue in the environment. It is the relationship between stimuli as cued by the pairing (i.e., pairings = similar) that determines the observed change in liking. Across five pre-registered studies (*N* = 747) we manipulated the absolute or relative distance between different pairs of conditioned (CS) and unconditioned stimuli (US) under the assumption that this would influence the type of relation that the pairings would cue (i.e., close = similar; far = different). In all five studies we obtained repeated and strong evidence that stimulus pairings led to changes in implicit and explicit evaluations. Although we found that these effects were moderated by absolute distance manipulations, evidence did not emerge indicating that those same effects were moderated by relative distance manipulations. These findings fail to provide strong support for a symbolic perspective on EC. We discuss the implications of our findings as well as future research in this area.

## Introduction

Evaluative Conditioning (EC) refers to a change in liking due to the pairing of stimuli, and is an important avenue via which evaluations can be established or changed. In a typical EC study a neutral conditioned stimulus (CS) acquires the valence of a positive or negative unconditioned stimulus (US) with which it was previously paired. For example, contiguous presentations of a neutral face with pleasant images can result in that person being evaluated positively whereas pairing the same individual with negative images results in them being evaluated negatively [1].

Received wisdom dictates that EC constitutes a “primitive” form of learning, presumably because the operation involved (the pairing of stimuli) is itself a simple one. Recently, however, this idea has been challenged by a new symbolic perspective on EC [2]. The symbolic perspective argues that, early on in their development, humans gain access to a symbolic learning pathway (i.e., they learn how to generate and use *symbols*). This ability enables them to imbue stimuli with symbolic meaning and humans constantly do so [3]. For instance, thousands of languages are spoken around the world every day consisting of individual symbols (words) that are strung together to convey complex meaning (sentences). Musical and mathematical notation systems have been constructed that also consist of abstract symbols. But symbols are not limited to words and notations. Physical gestures (such as a wink of the eye or a thumbs up) can convey as much meaning as a sentence or story. Roads are decorated with symbols indicating how people should behave (traffic signs), as are the insides of museums and art galleries (e.g., lines in front of paintings function as symbols telling people to stop), and the outsides of buildings, which are often decorated with symbols (e.g., lions, eagles, saints) dripping with specific meaning. In short, humans are surrounded by a rich variety of symbols and imbue stimuli with symbolic meaning each and every day.

Now if humans are capable of imbuing stimuli with symbolic meaning then they might also imbue environmental *regularities* with meaning. An environmental regularity refers to all states in the environment of the organism that entail more than the presence of a single stimulus or behaviour at a single moment in time [4]. Regularities can involve the repeated presentation of a single stimulus (e.g., as in mere exposure procedures) or relationships between stimuli and actions (e.g., as in approach-avoidance training procedures). We propose that stimulus pairings—the regularity at the core of EC—represents yet another regularity that can convey *relational symbolic meaning* (i.e., how one stimulus is related to another). Put simply, according to the symbolic perspective on EC, the pairing of stimuli changes liking because humans respond to those pairings as a contextual cue symbolizing that the CS and US are related in a certain way. EC research therefore provides unique information about the way in which liking changes as the result of symbolic meaning construction that is based on stimulus pairings [2].

### EC as a symbolic phenomenon

If pairings do function as a symbol indicating how stimuli are related, then two possibilities follow. First, there may be some ‘default’ symbolic meaning that people attribute to pairings when other relevant contextual information is missing. Drawing on past EC research [1] and work elsewhere in learning psychology [5], we believe that the ‘default’ symbolic meaning of pairings may be ‘similarity’–namely—that the CS and US are similar along a particular dimension (e.g., valence). Similarity relations typical lead to the assimilative effects seen in the EC literature wherein a CS acquires the same valence as a US. From this perspective, stimulus pairings may function in much the same way as the expression “*is similar to*” in the instruction “*A is similar to B”* functions (i.e., they symbolize that two stimuli share certain properties) [2]. Second, just as the symbolic meaning of individual stimuli can vary across contexts (e.g., the letter-string ‘beer’ refers to an animal [‘bear’] in Dutch and a beverage in English), it might also be the case that the symbolic meaning of stimulus pairings varies over contexts. In other words, it should be possible to change the type of relationship that pairings convey, from manipulating the context in which stimuli are paired [6], to priming [7], as well as the presence of verbal relational qualifiers [8], [9], instructions [10], [11], [12], [13], or the requirement to make on-line judgements [14].

The aforementioned symbolic perspective also leads to new predictions. For instance, if pairings do function as a symbol indicating that the CS and US are similar to one another, then manipulating the properties of pairings could impact how symbolically similar the CS and US are perceived to be—and by implication—how much the CS is liked or disliked. One such property is the *distance* between paired stimuli. One way to think about distance is in terms of a continuum along which stimuli can vary (from those that are relatively close to one another to those that are further apart). When viewed in this way we see that distance is a relative and relational concept (it only makes sense to say that something is close or far away in relation to another object or position along that continuum). Put another way, distance is a relational contextual cue (i.e., something in the environment that signals how two stimuli are related to one another along the distance continuum). Therefore just as a traffic light signals how people should behave (stop or go), distance signals how stimuli are related (close vs. far away). This might lead people to respond to those stimuli in a certain way (as being more or less similar to one another), and thus cause them to be liked or disliked to a greater or lesser extent.

Distance between stimuli can be manipulated in one of two ways. The first is simple and direct: it involves just two stimuli that differ in how close or far away they are from one another (for communication purposes we will refer to this as an *absolute distance manipulation*). The second way is more complex and indirect. The distance between the stimuli we are interested in is not directly but rather indirectly manipulated based on the relative distance between those stimuli and another pair of stimuli (for communication purposes we will refer to this as a *relative distance manipulation*). To illustrate, imagine participants complete an EC phase consisting of two types of trials: *focal* trials in which certain CSs and USs always appear at a medium distance from one another, and *filler* trials in which other CSs and USs appear relatively closer together or further apart. In a context where the filler pairs are presented further apart from each other, stimuli in the focal trials may be perceived as being closer together and thus more similar to one another. If so, then stronger assimilative EC effects should emerge for the focal compared to filler stimuli. Yet when the filler pairs are presented closer together, focal pairings are relatively speaking further apart (and thus may be considered as dissimilar to, or at least less similar than) the filler stimuli. If so, then EC effects for the focal stimuli should either be reversed or assimilative but weakened (depending on how participants symbolically construe the meaning of distance). The key point here is that, in both cases, distance may act as a relational contextual cue. But how that cue is manipulated varies: either simply and directly (in absolute distance manipulations) or in a more complex and indirect way (in relative distance manipulations).

We tested both of these ideas in five studies to see if they would moderate EC effects. In Experiment 1 we implemented an absolute distance manipulation during the EC phase, so that certain CSs and USs were presented close together whereas others were presented far apart. In Experiments 2–5 we implemented a relative distance manipulation during the EC phase similar to that mentioned above. Following the EC phase, CS evaluations were assessed via self-report ratings and an Implicit Association Test (IAT). We added an IAT as it is assumed to reflect more automatic instances of evaluation that can influence behavior in unique ways [15]. If the symbolic meaning of pairings can be altered through absolute distance manipulations (i.e., simple and direct manipulations), then we would expect to observe larger EC effects when the CS and US are presented close together than further apart. If that meaning can also be altered through relative distance manipulations (i.e., more complex and indirect manipulations) then we would expect to see larger (focal) EC effects when filler stimulus pairs are (relatively speaking) further apart from one another (and thus the focal stimuli are closer together) than when the filler stimuli are closer together (and thus the focal stimuli are further apart).

## Experiment 1

Our first experiment set out to investigate if an absolute distance manipulation would moderate the strength of explicit and implicit evaluations. Based on previous work, we anticipated that CS-US pairs presented close together would lead to stronger EC effects than CS-US pairs presented further apart [16].

### Method

#### Ethics statement

The Ethics Committee of the Faculty of Psychology and Educational Sciences at Ghent University granted ethical approval for the study procedures. All participants were assured that no harm would come to them in the process of experiment, and were told that this experiment involved a learning task, a speeded computer task, and self-reported questions. The results of all tests were kept confidential. Participants were informed that they had the right to stop the experiment at any time during the experiment. Written consent was obtained before the experiment began.

#### Participants and design

135 participants (78 women, *Mage* = 33.59, *SD* = 8.80) completed the study on the Prolific Academic website (https://prolific.ac) in exchange for a monetary reward (£1.50). The experiment was programmed in Inquisit 4.0 and hosted via Inquisit Web (Millisecond Software, Seattle, WA). It consisted of a 2 (*Stimulus Distance*: close vs. distant) x 2 (*Valence*: CS1[CS3]+US_positive_ vs. CS2[CS4]+US_negative_) between-subjects design. Self-reported ratings and IAT effects were the dependent variables. Three additional method factors were also manipulated across participants: stimulus identity (CS1 and CS3 vs. CS2 and CS4 assigned to positive US stimuli), evaluative task order (self-report or IAT first) and IAT critical block order (EC phase consistent vs. inconsistent first). The sample size was determined prior to data collection. Note that the study designs and data-analysis plans for all experiments were pre-registered and made available on the Open Science Framework website (https://osf.io/hdmek/). We report all manipulations and measures used in Experiments 1–5. All data were collected without intermittent data analysis. The data analytic plan, experimental scripts, and data are available at the above link.

### Materials

#### Stimuli

Four nonsense words (Morag, Cacht, Ailbe, Struan) served as CS1, CS2, CS3, CS4. USs consisted of six positive and six negative images selected from the Open Affective Standardized Image Set (OASIS) [17] (Positive image numbers: Dog 6, Lake 9, Fireworks 3, Penguins 2, Rainbow 2, Beach 1; Negative image numbers: War 1, Shot 3, Bloody Knife 1, KKK rally 1, Garbage dump 4, Scary face 1). The assignment of CSs to close or distant trials, or positive and negative USs was counterbalanced across participants.

### Procedure

Participants were first provided with a general overview of the experiment and then asked for their informed consent. Overall, the study consisted of four phases: US familiarization, EC, evaluative measures, and exploratory questions. The entire session took approximately 20 minutes.

#### US familiarization

Participants were first presented with each of the USs one at a time onscreen. This was to ensure that they were aware of the content of each image, given that the images were rather small due to the distance manipulation (*see below*). Thus each image was presented in a bigger size (30%) than in the EC phase (16%). The duration of each trial was 3000ms while the inter-trial interval (ITI) was 500ms.

#### EC

The following instructions were provided prior to the EC phase: “In the next part of the study you are going to encounter four new words (Morag, Ailbe, Struan, Cacht). You have probably never seen these words before. These words will appear together with an image. Important: some of the words and images are going to move away from one another. Other words and images will move towards one another. Pay attention to which words and images move away from or towards one another.”

**Fig 1:**
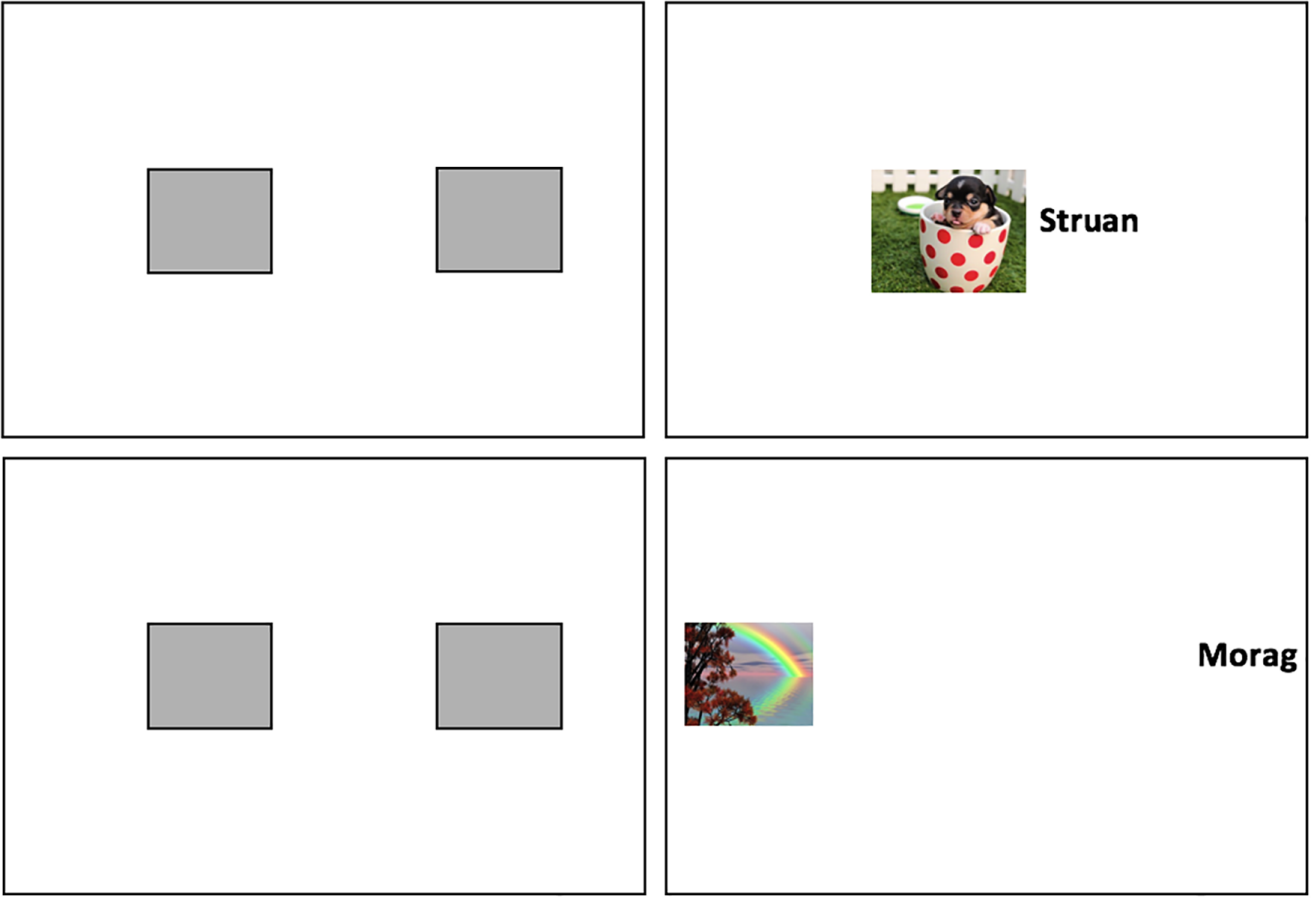
Examples of the close (*top right*) and distant (*bottom right*) trials as well as the starting point for each trial (medium distance; *top and bottom left* panels) in Experiment 1. Stimuli were initially hidden behind grey rectangles so that participants could process distance before contiguity.

The EC phase consisted of two blocks of 24 trials (48 total). Each trial began with two grey rectangles presented at a medium distance apart (stimulus coordinates on the horizontal axis were 30% and 70%, respectively). During half of the trials these two rectangles moved closer to one another, and once they were side-by-side, the rectangles disappeared to reveal CS1 or CS2 behind one and a positively or negatively valenced image (US) behind the other (stimulus coordinates on the horizontal axis at trial termination: 44% and 56%, respectively). On the other half of the trials the two rectangles moved away from one another, and once they were on opposite sides of the screen, the rectangles disappeared to reveal CS3 or CS4 behind one and a valenced image behind the other (stimulus coordinates on the horizontal axis at trial termination: 16% and 84%, respectively). CSs and USs remained onscreen together for another 1750ms. Thereafter all stimuli disappeared and the next trial began (see Fig 1).

#### IAT

Two IATs were administered: one to measure implicit evaluations of the CSs presented on ‘close’ trials (i.e., CS1 and CS2) and another to measure evaluations of CSs presented on ‘distant’ trials (i.e., CS3 and CS4). During both IATs participants were informed that two of the novel words they encountered during the learning phase (targets) as well as the words ‘Good’ and ‘Bad’ (attributes) would appear on the upper left and right sides of the screen, and that stimuli could be assigned to these categories using either the left (‘E’) or right keys (‘I’). If a word was correctly categorized, then the stimulus disappeared from the screen and the next trial began. In contrast, an incorrect response resulted in the presentation of a red ‘X’ which remained onscreen until the correct key was pressed. Overall, each IAT consisted of seven blocks of trials. The first block (20 practice trials) required them to sort the two nonsense words into their respective categories, with one word (e.g., CS1) assigned to the left (‘E’) key and the other (e.g., CS2) to the right (‘I’) key. On the second block (20 practice trials) participants assigned positively valenced stimuli to the ‘Good’ category using the left key and negative stimuli to the ‘Bad’ category using the right key. Blocks 3 (20 trials) and 4 (40 trials) involved a combined assignment of target and attribute stimuli to their respective categories. Specifically, participants categorized CS1 and ‘positive’ words using the left key and CS2 and ‘negative’ words using the right key. The fifth block (20 trials) reversed the key assignments, with CS1 now assigned to the right key and CS2 with the left key. Finally, the sixth (20 trials) and seventh blocks (40 trials) required participants to categorize CS1 with ‘negative’ words and CS2 with ‘positive’ words. A similar IAT was conducted with CS3 and CS4, and the order of the two IATs, as well as the critical test blocks in each IAT, was counterbalanced across participants.

#### Self-report measure

Stimulus ratings of the two stimuli presented close to (CS1 and CS2) or far away from the USs (CS3 and CS4) were obtained using a series of Likert scales. On each trial, participants were presented with a stimulus and asked to indicate whether they considered it to be ‘*Good/Bad*’, ‘*Pleasant/Unpleasant*’, ‘*Positive/Negative*’ and whether ‘*I like it/I don’t like it*’ using a scale ranging from -5 to +5 with 0 as a neutral point.

#### Exploratory questions

Participants were probed for CS-US *contiguity memory* (i.e., the extent to which they recalled the valence of the USs that CSs were paired with) and *distance memory* (i.e., the distance between CSs and USs). We also included a *manipulation check* to ensure that they did not write down the contingencies during the EC phase, along with a *hypothesis awareness*, *distance awareness and influence*, *demand compliance*, and a *reactance* question. They also completed a behavioral intention measure (as well as the Need for Cognition scale [NFC], [18] in Experiments 2–5). Note that many of these variables were included for exploratory purposes and will not be discussed further (for more see Supplementary Materials available at https://osf.io/hdmek/).

### Results

#### Data preparation

For explicit evaluations we calculated two difference scores—one for the CSs presented close to the USs (i.e., CS1 and CS2) and another for the CSs presented far away from the USs (i.e., CS3 and CS4). Positive values indicate a preference for the CS paired with positive images over the CS paired with negative images whereas negative values indicate the opposite response pattern. Following the recommendations of [19], response latency data were prepared using the D scoring algorithm. The resulting D IAT scores reflect the difference in mean response latency between the critical blocks divided by the overall variation in those latencies. IAT scores were calculated so that positive values reflected a response bias for the CS paired with positive stimuli (CS1 or CS3) relative to the CS paired with negative stimuli (CS2 or CS4) whereas negative values indicated a reverse response pattern. According to our analytic plan, IAT data were removed for participants who (a) had error rates above 30% when considering all IAT blocks or above 40% for any one of the critical IAT test blocks, or (b) responded faster than 400ms on more than 10% of the IAT trials (*n* = 4). We also excluded participants if they failed to complete the entire experimental session (*n* = 19). Note: we retained the data of participants who met the mastery criteria on one of the two IATs and discarded their data if they failed to meet those criteria on both IATs. This left a final sample of 112 participants.

#### Analytic plan

A series of paired-sample t-tests were carried out to determine whether implicit and explicit evaluations (*dependent variables*) differed as a function of Stimulus Distance (*close vs*. *distant*).

### Hypothesis testing

#### IAT

The IAT effect for stimuli presented close together (*M* = 0.11, *SD* = 0.43) did not differ from those that were presented further apart (*M* = 0.08, *SD* = 0.43), *t*(101) = 0.53, *p* = .60, *d* = 0.05, 95% CI [-0.14; 0.25], BF_01_ = 7.97. The IAT effect for stimuli presented close together significantly differed from zero, *t*(106) = 2.58, *p* = .01, *d* = 0.25, 95% CI [0.06; 0.44], BF_10_ = 2.50, unlike the IAT score for stimuli presented at a distance, *t*(106) = 1.88, *p* = .06, *d* = 0.18, 95% CI [-.01; 0.37], BF_01_ = 1.73.

#### Self-reported ratings

A paired-samples t-test revealed that the EC effect for the closely presented stimuli (*M* = 5.93, *SD* = 3.80) was significantly larger than that for the distantly presented stimuli (*M* = 3.16, *SD* = 5.39), *t*(111) = 5.11, *p* < .001, *d* = 0.48, 95% CI [0.29; 0.68], BF_10_ = 10039.27.

### Discussion

In-line with our initial hypothesis and previous findings [15], we found that the magnitude of explicit (but not implicit) EC effects can be altered via an absolute distance manipulation. CSs presented physically closer to USs led to stronger EC effects compared to those that were presented further away. This is despite the fact that the CS-US contingencies were identical for closely and distantly presented stimuli. In contrast, implicit evaluations of CSs presented close to USs did not differ from those presented further away. That said, IAT effects for stimuli presented close together did reach conventional levels of significance whereas IAT effects for stimuli presented at a distance did not. These initial findings support the idea that it is not merely the fact that stimuli are paired, but how they are paired, that drives EC effects.

## Experiment 2

In Experiment 2 we sought to demonstrate that EC effects can be moderated via relative distance manipulations. We now exposed participants to two types of EC trials: *focal* stimulus pairs (presented at a medium distance) and *filler* stimulus pairs that were (for some participants) presented closer together or (for other participants) presented further apart than the focal pairs. We hypothesized that the first group of participants would view the focal pairs as being relatively closer to one another (and thus more related) when filler pairs were presented at a larger distance. The second group might view the focal pairs as more distant (and thus less related) when the filler pairs were presented closer together. Overall, this should lead to larger implicit and explicit evaluations of the focal stimuli in the former compared to the latter group.

### Method

#### Participants and design

162 participants (94 women, *Mage* = 33.18, *SD* = 7.56) completed the study on the Prolific Academic website in exchange for a monetary reward (£1.50).

### Procedure

Experiment 2 was similar to Experiment 1 with the exception of the EC phase (*see below*). We also administered a single IAT measuring implicit evaluations of the focal CSs (i.e., CS1 and CS2) given that our interest was primarily in the extent to which we could change the symbolic meaning of distance for the focal (rather than filler) trials.

#### EC

**Fig 2:**
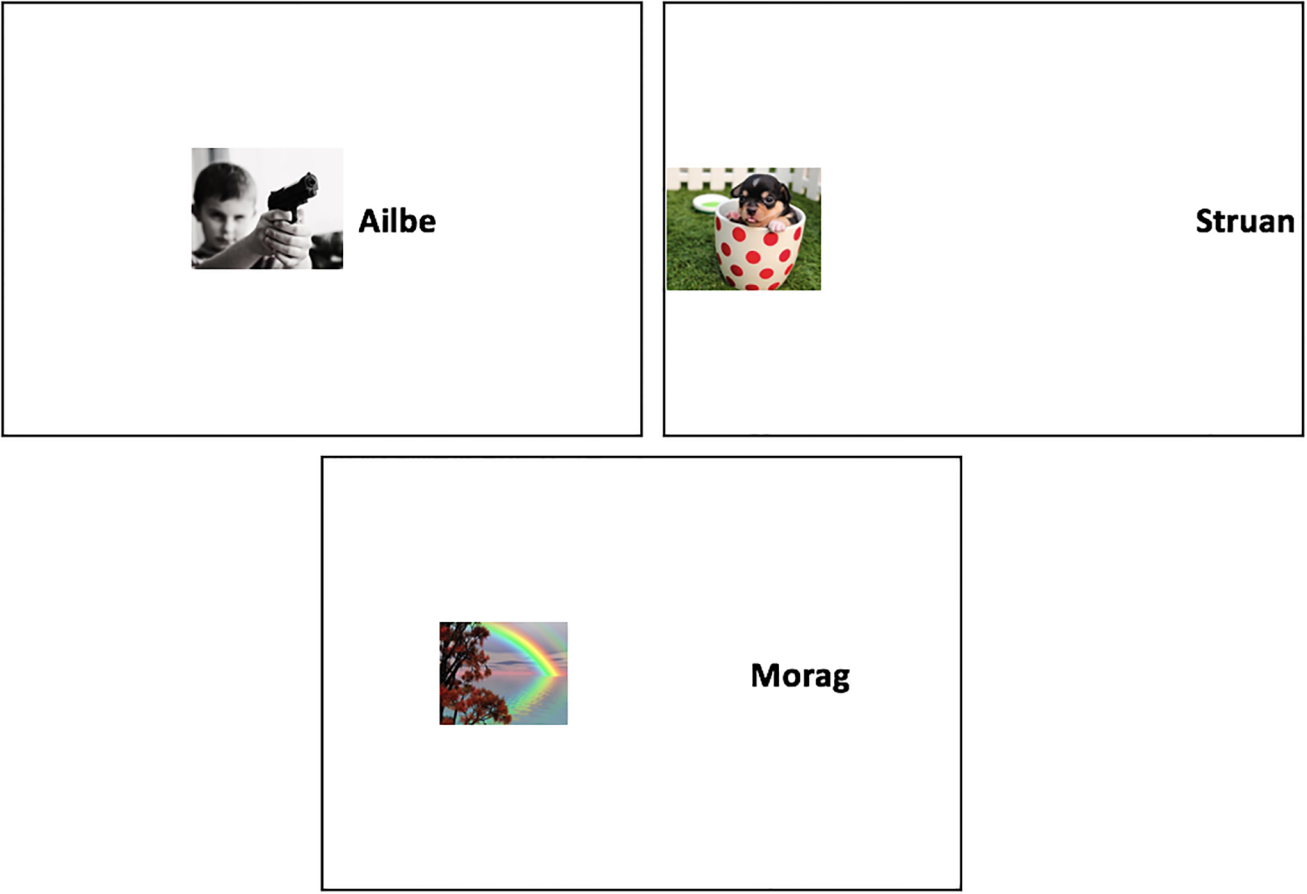
Examples of filler trials from the close (*top left*) and distant (*top right*) conditions as well as a focal trial at medium distance (*bottom centre*) in Experiment 2. Focal CSs and USs were always presented at a medium distance. Filler CSs and USs were presented relatively closer or further away from one another than the focal pairs.

EC consisted of four blocks of twenty trials (80 trials total). Each trial simultaneously presented either CS1, CS2, CS3 or CS4 on the left and a valenced image (USs) on the right side of the screen for 1750ms. In this phase each CS was paired with one of two different images of the same valence. Crucially, there were two types of trials. During focal trials, CS1 or CS2 was always presented on-screen at a medium distance from a US (CS and US coordinates on the horizontal axis of the screen: 30% and 70%, respectively). For those in the *large distance* condition, filler trials involved presenting CS3 or CS4 further away from a US (CS and US coordinates on the horizontal axis of the screen: 12% and 83%, respectively). For those in the *close distance* filler condition, filler trials involved presenting CS3 or CS4 close together with a US (CS and US coordinates on the horizontal axis of the screen: 43% and 61%, respectively). Note that, in both distance conditions, the CS and US appeared onscreen surrounded by a large rectangle. We included this to help participants recognize the relative distance between stimuli on focal and filler trials (see Fig 2).

### Results

#### Data preparation

For explicit evaluations we calculated a difference score for the focal stimuli (i.e., CS1 and CS2). An IAT score was calculated so that positive values reflected a bias for the focal CS paired with positive stimuli (e.g., CS1) over the focal CS paired with negative stimuli (e.g., CS2). Negative values indicated the reverse pattern of responding. A similar set of exclusion criteria were used as in Experiment 1. This left a final sample of 122 participants.

#### Analytic plan

A series of independent and paired samples t-tests were carried out to determine whether implicit and explicit evaluations of the focal stimuli (*dependent variables*) differed as a function of Relative Stimulus Distance (*close vs*. *distant*). Note that the same data preparation and analytic strategies were employed in all subsequent experiments.

### Hypothesis testing

#### IAT

For descriptive statistics see Tables 1 and 2. Submitting focal stimulus IAT scores (for those in the close and distant filler conditions) to an independent t-test did not reveal a significant difference, *t*(120) = -0.008, *p* = 0.99, *d* = -0.001, 95% CI [-0.36; 0.36], BF_01_ = 5.17. Participants in the close (*M* = 0.33, *SD* = 0.41), *t*(55) = 6.01, *p* < .001, *d* = 0.80, 95% CI [0.50; 1.10], BF_10_ > 10^3^ and distant conditions (*M* = 0.33, *SD* = 0.47), *t*(65) = 5.75, *p* < .001, *d* = 0.71, 95% CI [0.44; 0.98], BF_10_ > 10^3^ displayed a similar IAT score favoring the CS paired with positive over the CS paired with negative USs.

**Table 1:** Means and standard deviations for explicit and implicit evaluations of the focal stimuli in Experiments 2–5 as a function of Relative Stimulus Distance (i.e., distance of filler stimulus pairs relative to focal stimulus pairs).

	Self-Reported Ratings		IAT	
Filler Stimulus Distance	Close	Distant	Close	Distant
	*M SD*	*M SD*	*M SD*	*M SD*
Study 2	5.32 (5.11)	5.67 (4.57)	0.33 (0.41)	0.33 (0.47)
Study 3	5.40 (4.28)	5.33 (3.79)	0.26 (0.45)	0.31 (0.44)
Study 4	3.87(4.55)	3.87 (3.95)	0.14 (0.46)	0.34 (0.40)
Study 5	5.21 (4.19)	7.05 (3.17)	0.27 (0.40)	0.36 (0.42)

**Table 2:** Descriptive statistics for the CS-US Contingency awareness, Stimulus Distance Contingency, Distance Awareness, and Influence measures in Experiments 1–5.

	Study 1	Study 2	Study 3	Study 4	Study 5
CS-US Contingency Awareness	64 (57%)	86 (71%)	88 (60.7%)	56(46%)	87 (73%)
Stimulus Distance Contingency	64 (57%)	8 (6.6%)	92 (63.4%)	83 (68%)	64 (54%)
Stimulus Distance Awareness	67 (60%)	56 (45.9%)	109 (75.2%)	78 (63%)	71 (60%)
Stimulus Distance Influence	42 (38%)	24 (19.7%)	42 (29%)	48 (39%)	25 (21%)

Participant were said to have passed the CS-US Contingency Awareness and Stimulus Distance Contingency measures if they accurately recalled all four contingencies in either case. Stimulus Distance Awareness refers to the percentage of participants who were aware that filler stimulus distance was manipulated during the EC phase whereas Stimulus Distance Influence refers to the percentage of participant who indicated that the distance information influenced their CS evaluations.

#### Self-reported ratings

Submitting self-report ratings of the focal stimuli to a similar t-test did not reveal any difference as a function of Stimulus Distance, *t*(120) = -0.40, *p* = 0.69, *d* = -0.07, 95% CI [-0.43; 0.28], BF_01_ = 4.80. Participants showed similar and strong (focal) EC effects in the close, *t*(55) = 7.79, *p* < .001, *d* = 1.04, 95% CI [0.71; 1.36], BF_10_ > 10^4^, and distant conditions, *t*(65) = 10.08, *p* < .001, *d* = 1.24, 95% CI [0.92; 1.56], BF_10_ > 10^4^.

### Exploratory analyses

#### Self-reports

Although we were primarily interested in the impact of relative distance on focal stimulus evaluations we also examined if those same manipulations would influence evaluations of the filler stimuli.

Submitting self-report ratings of the filler stimuli to an independent samples t-test did not reveal a main effect for Stimulus Distance, *t*(120) = -0.04, *p* = .97, BF_01_ = 5.16. Participants showed similar strong (filler) EC effects in the close (*M* = 5.87, *SD* = 4.89), *t*(55) = 8.98, *p* < .001, *d* = 1.20, 95% CI [0.85; 1.54], BF_10_ > 10^4^, and distant conditions (*M* = 5.90, *SD* = 4.82), *t*(65) = 9.94, *p* < .001, *d* = 1.22, 95% CI [0.90; 1.54], BF_10_ > 10^4^. A 2 (*Stimulus Type*; focal vs. filler) x 2 (*Stimulus Distance*) ANOVA revealed no main effect for Stimulus Type, *F*(1, 120) = 1.79, *p* = .18, BF_01_ = 3.32, or Distance, *F*(1, 120) = 0.06, *p* = .82, BF_01_ = 2.84, nor a significant interaction between the two, *F*(1, 120) = 0.30, *p* = .59, BF_01_ = 5.07.

### Discussion

Although we successfully induced implicit and explicit evaluations towards focal and filler CSs, we failed to find evidence that EC effects were moderated by a relative distance manipulation. Upon closer inspection it appears that most participants were not aware that the distance between CSs and USs actually differed on the filler trials. It may be that a stronger manipulation is necessary in order to increase awareness that distance is actually changing in such indirect manipulations. We explored this idea in Experiment 3.

## Experiment 3

**Fig 3:**
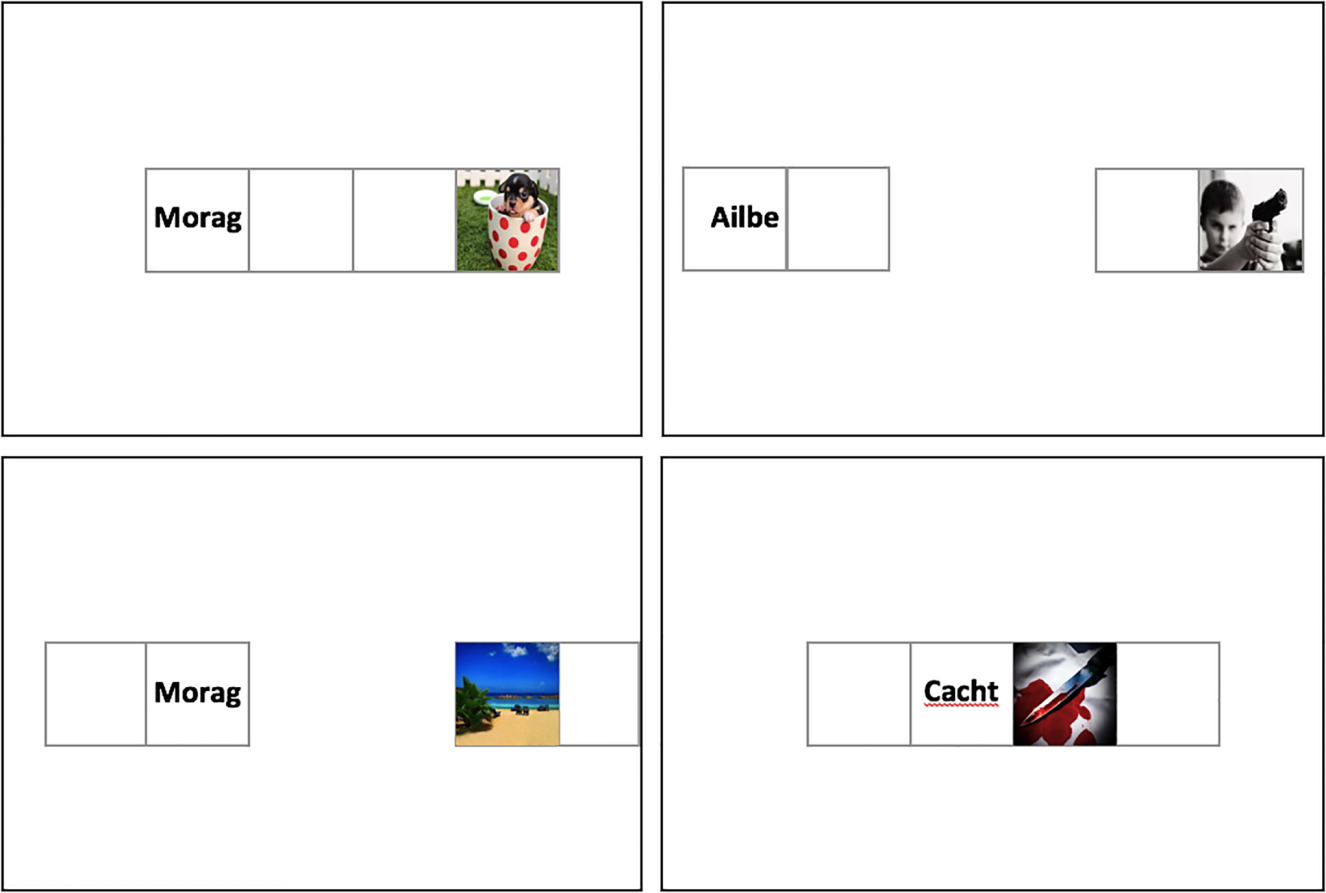
Examples of filler trials from the close (*bottom right*) and distant (*top right*) conditions as well as their corresponding focal trials at medium distance (*left panels*) in Experiment 3. Focal CSs and USs were always presented at a medium distance. Filler CSs and USs were presented relatively closer or further away from one another than the focal pairs.

In Experiment 3 we sought to heighten participant’s awareness of the fact that filler and focal stimulus pairs differed in their relative distances, and in so doing, examine if this moderated focal stimulus evaluations. A similar setup was used as in Experiment 2 with three exceptions, all designed to increase awareness of the relative distance manipulation. First, the EC phase was now preceded by *instructions* indicating that stimuli would be paired, and that in some cases stimuli would be presented close together, whereas in others they would be presented far away from one another (similar to Experiment 1). Second, we presented *four rectangles* onscreen during each EC trial to emphasize the different location of stimuli (see Fig 3). CSs and USs appeared in two of the four rectangles, depending on whether they were either focal or filler stimuli. Third, we decided to remove the large grey rectangle as this may have undermined our previous distance manipulation.

### Method

#### Participants and design

174 participants (81 women, *Mage* = 32.61, *SD* = 8.35) took part via Prolific Academic in exchange for a monetary reward. Stimuli, experimental design, dependent variables, and exploratory questions mirrored those adopted in Experiment 2. Only the EC phase differed.

#### EC

The EC phase was now preceded by instructions indicating that: “In the next part of the study a word and an image will appear onscreen. Words will appear on the left and images on the right side of the screen. Important: certain words and images will appear close together. Others will appear far away from each other. It is important that you pay careful attention to which words and images are close together or far apart because we will ask you questions about this later on.”

To further emphasize that distance varied across filler and focal trials, four rectangles were now presented during each trial. The location of these rectangles varied depending on the distance condition: in the *distant* condition, the four rectangles were located at the extreme and the medium left and right part of the screen, while in the *close* condition the rectangles were located at the medium and the inner left and right part of the screen. A CS and a US could appear in either the medium or in the extreme outer or inner rectangles, leaving the other two rectangles blank. Finally, and unlike Experiment 1, no large rectangle surrounded the stimuli onscreen (see Fig 3).

### Results

#### Data preparation

A similar set of exclusion criteria were used as in Experiment 2. This left us with a final sample of 145 participants.

### Hypotheses testing

#### IAT

Analyses did not reveal a difference in scores as a function of Stimulus Distance, *t*(144) = -0.71, *p* = 0.48, *d* = -0.12, 95% CI [-0.44; 0.21], BF_01_ = 4.40, with participants in the close, *t*(68) = 4.66, *p* < .001, *d* = 0.56, 95% CI [0.31; 0.81], BF_10_ = 1192, and distant conditions, *t*(76) = 6.17, *p* < .001, *d* = 0.70, 95% CI [0.45; 0.95], BF_10_ > 10^4^, both showing strong and similar IAT scores.

#### Self-reported ratings

Submitting self-report ratings for the focal stimuli to a similar t-test did not reveal a significant difference as a function of Stimulus Distance, *t*(144) = 0.10, *p* = 0.92, *d* = 0.02, 95% CI [-0.31; 0.34], BF_01_ = 5.57. Participants showed similar and strong focal EC effects in the close, *t*(68) = 10.35, *p* < .001, *d* = 1.25, 95% CI [0.93; 1.56], BF_10_ > 10^4^, and distant conditions, *t*(76) = 12.35, *p* < .001, *d* = 1.41, 95% CI [1.09; 1.72], BF_10_ > 10^4^.

#### Exploratory analyses

Submitting self-report ratings for the filler stimuli to a similar analyses as reported above did not reveal a main effect for Stimulus Distance, *t*(1, 143) = 1.68, *p* = .10, BF_01_ = 1.54. Participants showed similar filler EC effects in the close (*M* = 6.57, *SD* = 3.75), *t*(68) = 14.30, *p* < .001, *d* = 1.72, 95% CI [1.35; 2.09], BF_10_ > 10^4^, and distant conditions (*M* = 5.50, *SD* = 3.87), *t*(76) = 12.48, *p* < .001, *d* = 1.42, 95% CI [1.10; 1.1.74], BF_10_ > 10^4^. A 2 (*Stimulus Type*; focal vs. filler) x 2 (*Stimulus Distance*) ANOVA revealed a main effect for Stimulus Type, *F*(1, 143) = 4.18, *p* = .04, *η*^*2*^_*p*_ = .03, BF_10_ = 0.76. There was no main effect for Distance, *F*(1, 143) = 1.01, *p* = .32, BF_01_ = 3.11, nor a significant interaction between the two, *F*(1, 143) = 2.33, *p* = .13, BF_01_ = 1.92.

Interestingly, when we compared EC effects for the focal stimuli (presented at a medium distance) to the filler stimuli that were presented relatively closer to one another, we did find that focal stimuli effects were smaller (*M* = 5.34, *SD* = 4.28) than those for the filler stimuli (*M* = 6.49, *SD* = 3.77), *t*(68) = 2.20, *p* = .03, *d* = 0.27, 95% CI [0.02; 0.51], BF_10_ = 1.27 (note however, that the value of this Bayes Factor was sensitive to our choice of prior [Cauchy = .707] and is contingent on the available data). Yet when we compared the EC effects for the focal stimuli (at a medium distance) to the filler stimuli (at a large distance) we found no such difference, *t*(76) = 0.43, *p* = .67, BF_01_ = 7.29.

### Discussion

Similar to Experiment 2, we induced strong implicit and explicit evaluations towards focal CSs, and once again failed to find evidence that these effects were moderated by relative distance manipulations. Exploratory analyses did reveal an impact of absolute distance on evaluations when considering medium focal vs. close filler stimuli: evaluations were smaller for the focal stimuli (presented at a medium distance) than for filler stimuli presented relatively closer together.

## Experiment 4

The relative distance manipulations used in Experiments 2–3 required participants to discern the relative distance between focal and filler CS-US pairs by comparing one type of trial (filler) to another (focal) (i.e., engage in a cross trial-type comparison). In Experiment 4 we sought to make this comparison even easier by making distance changes as salient as possible. Specifically, we now used *movement* during the filler trials to convey that the distance between CSs and USs was increasing or decreasing, and a lack of movement on the focal trials to signal that no such distance change was taking place (similar to Experiment 1). This time every stimulus started from the same position. Whereas the focal CSs never moved and simply remained static for the duration of the trial, the filler CSs either moved closer together (*close* condition) or further apart (*distant* condition). In this way we hoped to increase the probability that people would incorporate distance information when subsequently making a CS evaluation. Once again, our reasoning was that focal CSs should be evaluated more positively or negatively than the filler CSs in the distant condition (given that—in comparison—the focal stimuli are physically closer to each other) and less positively or negatively than the filler CSs in the close condition (given that—in comparison—the focal stimuli are physically more distant to one another).

### Method

#### Participants and design

139 participants (94 women, *Mage* = 32.05, *SD* = 7.83) took part via Prolific Academic for a monetary reward. Stimuli, experimental design, dependent variables, and exploratory questions mirrored Experiments 2–3. Only the EC phase differed.

#### EC

The following instructions were provided: “In the next part of the study a word and an image will appear onscreen. Words will appear on the left and images on the right side of the screen. Important: some pairs will remain far apart whereas other pairs will move close together. It is important that you pay careful attention to which words and images remain far apart [close together] or move close to [far away from] each other. We will ask you questions about this at the end of the study”.

**Fig 4:**
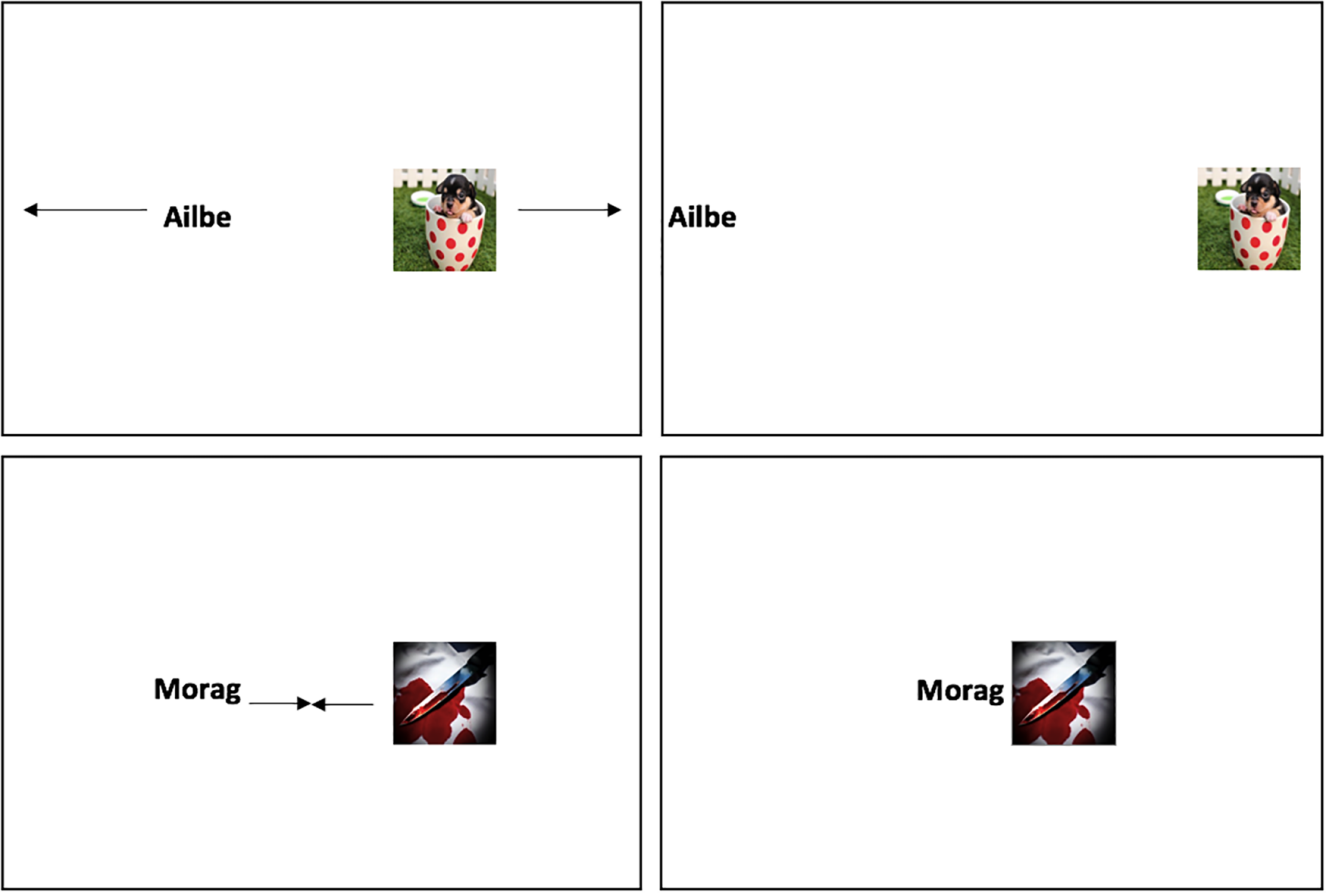
Examples of filler trials from the distant (*top right*) and close (*bottom right*) conditions as well as the corresponding starting positions of those trials (*top and bottom left panels*) in Experiment 4. Focal CSs and USs were always presented at a medium distance. Filler CSs and USs were presented relatively closer or further away from one another than the focal pairs.

The EC procedure consisted of three blocks of twenty-four trials. Trial duration was fixed across each type of trial (focal and filler), and increased (4000ms) to give participants enough time to process the movement of the stimuli onscreen in the filler trials. Each trial simultaneously presented either CS1, CS2, CS3 or CS4 on the left and valenced images (USs) on the right for 1750ms. On focal trials, CS1 or CS2 remained at a medium distance from the US (stimulus coordinates on the horizontal axis were 30% and 70%, respectively). During the distant filler trials, CS3 or CS4 also began at the medium distance. After 1750ms they moved along a horizontal axis in the opposite direction to the US (stimulus coordinates on the horizontal axis at trial termination: 16% and 84%, respectively). During close filler trials, the CS was initially located at the same (medium) distance as the focal stimuli. After 1750ms the CS and US moved towards each other (stimulus coordinates on the horizontal axis at trial termination: 44% and 56%, respectively) (see Fig 4).

### Results

#### Data preparation

Participants who did not complete the entire session or who failed to meet the IAT criteria were excluded from analyses (*n* = 16). This left a final sample of 123 participants.

#### IAT

Analyses revealed a difference in scores as a function of Stimulus Distance, *t*(122) = -2.57, *p* = 0.01, *d* = -0.47, 95% CI [-0.82; -0.11], BF_10_ = 3.67. IAT scores for the focal stimuli were smaller when the filler stimuli were relatively closer and the focal stimuli relatively further away from one another, (*M* = 0.14, *SD* = 0.46), *t*(65) = 2.44, *p* = .02, *d* = 0.30, 95% CI [0.05; 0.55], BF_10_ = 2.13. Those same scores were larger whenever the filler stimuli were relatively further away and the focal stimuli closer together, (*M* = 0.34, *SD* = 0.40), *t*(56) = 6.42, *p* < .001, *d* = 0.85, 95% CI [0.54; 1.15], BF_10_ > 10^4^.

#### Self-reported ratings

Analyses did not reveal a difference in focal stimulus ratings as a function of Stimulus Distance, *t*(122) = -0.01, *p* = 0.99, *d* = -0.001, 95% CI [-0.36; 0.35], BF_01_ = 5.19. Participants showed similar and strong (focal) EC effects in the close, *t*(65) = 6.91, *p* < .001, *d* = 0.85, 95% CI [0.57; 1.13], BF_10_ > 10^4^, and distant conditions, *t*(56) = 7.41, *p* < .001, *d* = 0.98, 95% CI [0.66; 1.30], BF_10_ = 10^4^.

#### Exploratory analyses

Submitting filler stimulus ratings to a similar set of analyses did not reveal a main effect of Stimulus Distance, *t*(121) = 1.82, *p* = 0.07, BF_01_ = 1.17 (note however, that the value of this Bayes Factor was sensitive to our choice of prior [Cauchy = .707] and is contingent on the available data). A 2 (*Stimulus Type*) x 2 (*Stimulus Distance*) ANOVA revealed a main effect for Stimulus Type, *F*(1, 121) = 9.60, *p* = .002, *η*^*2*^_*p*_ = .07, 95% CI [0.01; 0.17], BF_10_ = 15.39, such that EC effects were larger for the filler compared to the focal stimuli. No such main effect was found for Stimulus Distance, *F*(1, 121) = 1.15, *p* = 0.29, BF_01_ = 2.76. Marginally significant evidence also emerged for a two-way interaction between Stimulus Type and Distance, *F*(1, 121) = 3.28, *p* = .07, *η*^*2*^_*p*_ = .03, 95% CI [0.00; 0.10], BF_10_ = 0.83. Similar to Experiments 2–3, when we compared EC effects of the focal stimuli (presented at a medium distance) to the filler stimuli that were presented relatively closer to one another, we found that focal stimulus effects were smaller (*M* = 3.87, *SD* = 4.55) than the filler stimuli (*M* = 5.84, *SD* = 4.21), *t*(65) = 3.59, *p* = .001, *d* = 0.44, 95% CI [0.19; 0.69], BF_10_ = 38.70. When we compared the EC effects for the focal stimuli to the filler stimuli that were presented relatively further apart, no such difference emerged, *t*(56) = 0.88, *p* = .38, BF_01_ = 4.77.

### Discussion

Experiment 4 provided evidence that implicit EC effects can be moderated by relative distance manipulations. Changing the EC phase so that filler stimuli now moved closer or further away from one another had an impact on implicit evaluations of the focal stimuli. Specifically, IAT scores for the focal stimuli were stronger whenever the filler stimuli moved further away from each other (and focal stimuli remained closer together) than when they moved close together (and focal stimuli remained far apart). Yet we did not find an impact of relative distance on explicit EC effects. Similar to Experiments 2–3, exploratory analyses revealed evidence for an absolute distance effect, such that stronger EC effects emerged for filler CSs that were relatively closer to USs than focal CSs at a medium distance from the US.

## Experiment 5

Upon reflection, the EC phase in Experiments 2–4 confronted participants with two pieces of information: that the CSs and USs were presented contiguously with one another, and that the relative distance between pairs of stimuli could vary. Although instructions highlighted that distance (and not just mere contiguity) was task relevant, the EC phase itself did not *require* individuals to process the relative distance between stimulus pairs at any point. Indeed, it seems that many participants simply focused on contiguity and ignored distance: in Experiments 2–4 participants registered the contiguity between the CS and US and only sometimes noticed that pairs of stimuli could differ in their relative distance from one another. Thus it may be that relative distance is more likely to moderate EC effects when such information is provided prior to contiguity information than after it.

Towards this end we altered the EC phase so that (similar to Experiment 1) two grey rectangles initially appeared onscreen. During the focal trials these rectangles did not move. During filler trials they either moved closer or further apart. After a period, the grey rectangles disappeared and the CS and US took their place. In this way we hoped participants would initially process the distance information and only afterwards consider that valenced and non-valenced stimuli were presented in contiguity with one another.

### Method

#### Participants and design

137 participants (84 women, *Mage* = 33.94, *SD* = 8.63) completed the study via Prolific Academic in exchange for a monetary reward.

#### EC

The following instructions were provided: “In the next part of the study you are going to learn about four new words: Morag, Ailbe, Struan, Cacht. You have probably never encountered these words before. These words will appear individually onscreen together with some images. We are going to initially hide the word and image behind two grey rectangles. Later on we will reveal what was behind the two rectangles. Important: some of the words and images are going to remain far apart from [close together to] one another. Others will move closer together to [far away from] each other. It is important that you pay attention to the words and images that remain far apart [close together] or move closer together to [far away from] each other. We will ask you questions about this at the end of the study”.

**Fig 5:**
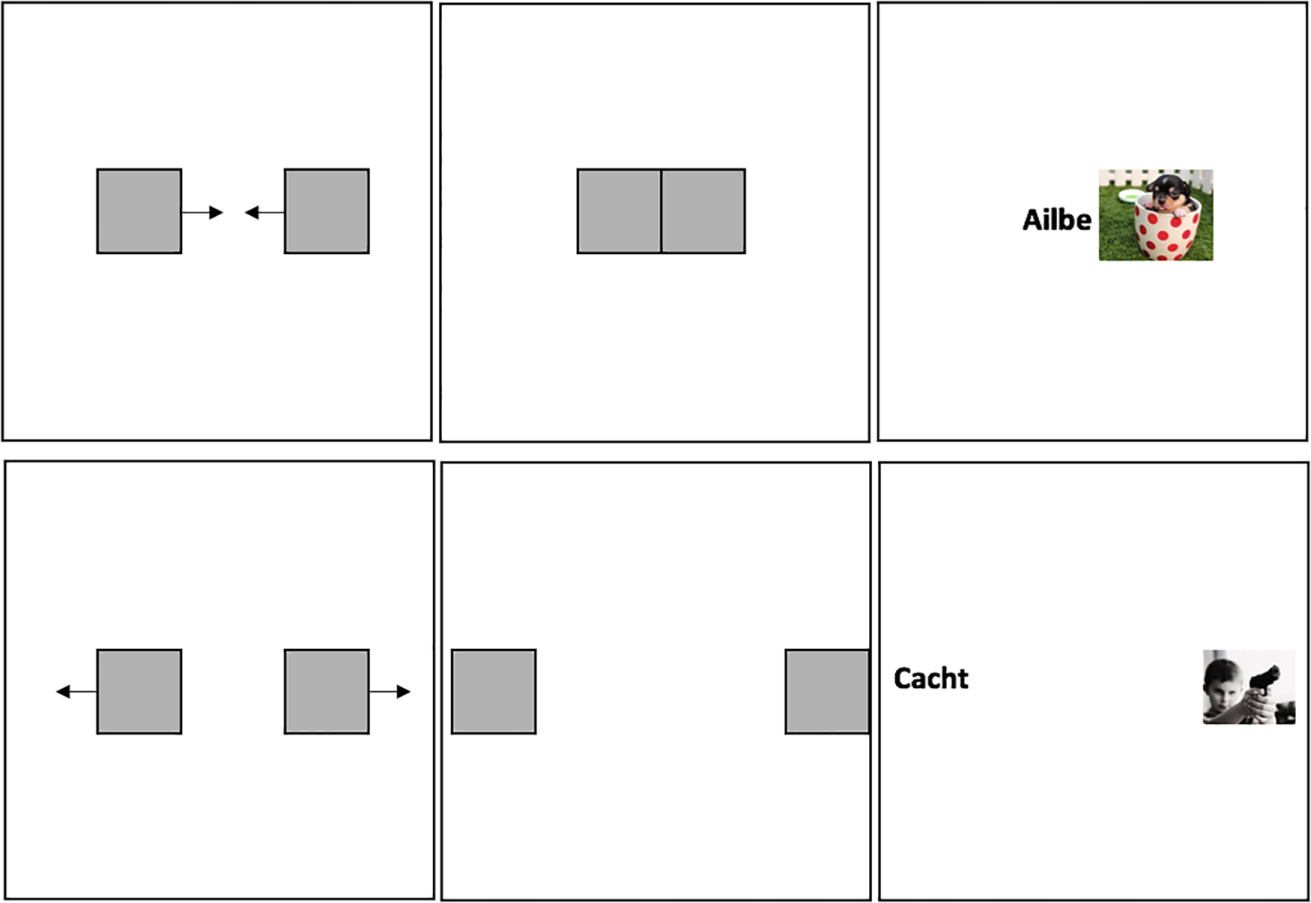
Examples of filler trials from the close (*top panels*) and distant (*bottom panels*) conditions as well as the corresponding starting positions of those trials (*far left panels*) in Experiment 5. Focal CSs and USs were always presented at a medium distance. Filler CSs and USs were presented relatively closer or further away from one another than the focal pairs.

The EC procedure was identical to that implemented in Experiment 4 with the following exceptions. All trials now started with the CS and the US covered by two grey rectangles for 2200ms. Thereafter the grey rectangles disappeared and the CS appeared from behind one and a valenced image (US) appeared from behind the other. These stimuli remained onscreen together for another 1750ms. Thereafter all stimuli disappeared and the next trial began (see Fig 5).

### Results

#### Data preparation

Application of the exclusion criteria led to the removal of eighteen individuals and a final sample of 119 participants.

### Hypothesis testing

#### IAT

Analyses did not reveal a difference in scores as a function of Stimulus Distance, *t*(117) = -1.25, *p* = 0.22, *d* = -0.23, 95% CI [-0.59, 0.13], BF_01_ = 2.54, with participants in the close, *t*(55) = 4.99, *p* < .001, *d* = 0.67, 95% CI [0.37; 0.95], BF_10_ > 10^3^, and distant filler conditions, *t*(62) = 6.74, *p* < .001, *d* = 0.85, 95% CI [0.56; 1.14], BF_10_ > 10^4^, both showing strong and similar IAT scores.

#### Self-reported ratings

Analyses on focal stimulus ratings revealed a main effect of Stimulus Distance, *t*(117) = -2.72, *p* = 0.007, *d* = -0.50, 95% CI [-0.87; -0.13], BF_10_ = 5.23. Stronger EC effects for the focal stimuli emerged whenever those stimuli were relatively closer together than the filler stimuli, *t*(62) = 17.67, *p* < .001, *d* = 2.23, 95% CI [1.76; 2.69], BF_10_ > 10^4^, and smaller when the focal stimuli were relatively further apart than the filler stimuli, *t*(55) = 9.29, *p* < .001, *d* = 1.24, 95% CI [0.89; 1.59], BF_10_ > 10^4^.

#### Exploratory analyses

Submitting the filler stimuli to similar analyses as reported above revealed no main effect of Stimulus Distance, *t*(117) = 1.46, *p* = .15, BF_01_ = 1.97. A 2 (*Stimulus Type*) x 2 (*Stimulus Distance*) ANOVA revealed no main effect for Stimulus Type, *F*(1, 117) = 1.14, *p* = .29, BF_01_ = 5.16, or Stimulus Distance, *F*(1, 117) = 0.31, *p* = .58, BF_01_ = 4.75. However, a two-way interaction between Stimulus Type and Distance did emerge, *F*(1, 117) = 12.05, *p* = .001, η^2^_partial_ = .09, 95% CI [0.02; 0.20], BF_10_ = 42.80. Similar to Experiments 2–4, when we compared EC effects of the focal stimuli (presented at a medium distance) to the filler stimuli that were presented relatively closer to one another, we found that focal stimulus effects were smaller (*M* = 5.21, *SD* = 4.19) than the filler stimuli (*M* = 7.18, *SD* = 3.21), *t*(55) = 3.83, *p* < .001, *d* = 0.51, 95% CI [0.23; 0.79], BF_10_ = 75.14. When we compared the EC effects for the focal stimuli, (*M* = 7.05, *SD* = 3.17) to the filler stimuli that were presented relatively further apart (*M* = 6.00, *SD* = 5.21), no such difference emerged, *t*(62) = 1.54, *p* = .13, BF_01_ = 2.38.

### Discussion

Altering the EC phase so that participants now had to process relative distance prior to contiguity moderated explicit (but not implicit) evaluations. Specifically, focal stimulus ratings were stronger whenever the filler stimuli moved further away from each other (and focal stimuli remained closer together) than when they moved closer together (and focal stimuli remained far apart). Exploratory analyses once again revealed evidence for an absolute distance effect such that filler CSs closer to USs were evaluated more strongly than focal CSs at a medium distance from USs.

#### Meta-analysis of Experiments 2–5

In order to provide a more robust estimate as to whether EC effects are moderated by relative distance manipulations, we conducted a mini meta-analysis based on the data from Experiments 2–5 following the practice proposed by [20]. Experiment 1 was excluded from this analysis given that it involved a within- rather than between-subjects design that was not directly comparable to the other experiments. Meta-analyses revealed that across studies relative distance did not influence explicit focal stimulus ratings as illustrated by a mean weighted effect size of *d* = -0.13, Z = -1.41, *p* = 0.16. A Bayesian one-way ANOVA with Stimulus Distance as a fixed factor (and study ID as a random factor) further supported this conclusion, such that focal ratings in the close (*M* = 4.93, *SD* = 4.55) and distant filler trials (*M* = 5.51, *SD* = 4.03) were not found to differ from one another across studies, BF_01_ = 3.99. In contrast, relative distance appears to have influenced implicit evaluations towards the focal stimuli, *d* = -0.20, Z = -2.24, *p* = .03. However, a similar Bayesian one-way ANOVA as reported above suggests that only anecdotal evidence emerged supporting the idea that IAT scores differed across studies in the close (*M* = 0.24, *SD* = 0.44) and distant filler conditions (*M* = 0.33, *SD* = 0.43), BF_10_ = 1.09.

## General discussion

We recently proposed a new symbolic perspective on EC that draws on the following ideas: that (a) pairings constitute a relational contextual cue in the environment, (b) humans treat this cue as a symbol indicating that the CS and US are related in a certain way, and (c) it is the symbolic relationship between stimuli—established by pairings—which determines the subsequent change in liking. An idea which follows from this perspective is that if one were to manipulate the properties of pairings then this could influence how much pairings function as a symbolic cue—and as a result—influence resulting changes in liking. A core property of pairings is the physical distance between stimuli, and the meaning of distance can potentially be manipulated in two ways. The first (absolute distance manipulations) is simple and direct: it involves just two stimuli that differ in how close or far away they are from one another. The second (relative distance manipulations) is more complex and indirect. It involved two types of trials: *focal* trials (in which a CS and US were always presented at a medium distance) and *filler* trials (in which other CSs and USs were presented closer together or further apart). Presenting filler stimuli far away from one another meant that focal stimuli were—by comparison—relatively closer together (and thus could be seen as more similar to one another). Presenting filler stimuli close together meant that the focal stimuli were—by comparison—relatively further apart (and thus might be seen as less similar to one another). If so, then we should observe larger EC effects in the former compared to latter scenario.

In Experiments 1–5 we obtained repeated and strong evidence for an impact of stimulus pairings on implicit and explicit evaluations of the focal stimuli (i.e., we always observed EC effects). We also obtained evidence that those same effects could be moderated by distance manipulations, but only for one type (absolute) and less so for another (relative). Manipulating the *absolute* distance between two stimuli influenced explicit evaluations, such that EC effects were larger whenever CSs and USs were physically closer than far apart. We obtained these effects in four of our five studies, suggesting that people can and do evaluate CSs differently depending on their absolute distance from the US. Note that one of our experiments also tested for the impact of absolute distance on implicit evaluations (Experiment 1). Although IAT effects for the close and distant conditions did not significantly differ from one another, only those for the closely presented stimuli (and not the distantly presented stimuli) differed from zero.

In contrast, manipulating distance in a relative fashion was largely ineffective. In Experiment 3, for instance, a relative distance manipulation moderated implicit evaluations (i.e., IAT effects were stronger when focal were closer together and smaller whenever they were further apart than filler pairings). Yet these effects were only obtained in one of our five studies: in all other cases relative distance failed to impact IAT scores. Likewise, in Experiment 5, we found that relative distance moderated explicit evaluations (i.e., stronger EC effects when focal pairs were relatively closer together and smaller when they were further apart than filler pairs). Yet, once again, this effect only emerged in one of our five studies, limiting the conclusions that can be drawn. This was further reinforced by our meta-analysis of Experiments 2–5 where relative distance was not found to moderate EC effects.

### Theoretical implications

#### Non-symbolic perspectives

One could interpret our findings in several ways. Consider a non-symbolic account of EC. This account would argue that pairings do not function as a symbolic cue and are a mere proximal cause of changes in liking. Our inability to moderate EC effects via relative distance manipulations in Experiments 2–5 could be seen as support for such a perspective. Likewise, findings from the non-human learning literature (with organisms that likely lack the ability to learn symbolically) suggest that classical conditioning effects can be moderated by the absolute distance between a CS and US; [21], [22]). Thus it may be that even the absolute distance effects observed here were non-symbolic in nature. Although such an account of EC is certainly plausible it is not without its problems. It rests on the assumption that pairings always function as a mere proximal cause of liking and never as a symbolic cue. Yet recent work indicates that the meaning of pairings can be altered via instructions, relational qualifiers, priming, online judgements, and contextual manipulations (e.g., [8], [10], [11], [12], [13], [14]). Thus a non-symbolic account of EC can accommodate our findings but not the wider trend of evidence elsewhere in the literature.

#### Symbolic perspectives

The failure to find an impact of relative distance on EC effects is inconsistent with the symbolic account we forwarded in the introduction. We assumed that one way of manipulating the meaning of pairings (relative distance) would be similar to others used in the literature (verbal information). This was clearly not the case. Thus the question becomes: why did absolute distance manipulations have an impact on EC effects whereas relative manipulations did not? The symbolic account could accommodate these findings in two ways. The first (a *strong symbolic* account) would argue that pairings are always a symbolic cue [for organisms with the ability to learn symbolically] and that relative distance was simply too weak a way of changing the meaning of that cue). In other words, spatiotemporal contiguity may already be a powerful relational cue for similarity, and to manipulate the meaning of that cue, one has to operate on it directly (change the absolute distance between two stimuli) rather than indirectly (change the relative distance between different pairs of stimuli and hope that participants recognise such a difference). This assumption could be reconciled with our results: despite repeatedly telling people that relative distance was an important cue to pay attention to, and physically moving those stimuli onscreen, many people simply disregarded this information, attended to the contiguity between stimuli, and relied on that mere contiguity when forming their evaluations. It also places a boundary condition on a strong symbolic account by showing that certain interventions (e.g., verbal information) are better able to change the meaning of a symbolic cue than others (relative distance). An alternative (*weak symbolic* account) is that pairings are initially a mere cause of changes in liking but the addition of new information (e.g., about distance) can transform it into a symbolic cue. This would explain moderation by absolute distance and the absence of that moderation by relative distance (if one assumes that absolute distance is a stronger method of changing the meaning of pairings than relative distance). Either way, the current findings are often inconsistent with the symbolic account we forwarded in the introduction and place constraints on that account going forward.

#### Alternative perspectives

The current findings are also compatible with other theoretical perspectives. Take the implicit misattribution model [16] which argues that EC effects result from the tendency for people to mistakenly (and unknowingly) attribute the valence evoked by the US to the (simultaneously presented) CS, thus leading to a change in how the CS is subsequently evaluated. The authors argue that misattribution is more likely to occur as ‘source confusion’ increases (i.e., as people confuse the multiple, contiguous elements in the environment that influence the likelihood that an evaluation of the US is misattributed to the CS). One variable argued to increase source confusion is the distance between stimuli. Presenting stimuli closely together is argued to increase the likelihood that they are processed in close temporal contiguity (thereby ensuring that CS and US representations are activated simultaneously). Conversely, presenting stimuli at a distance increases the likelihood that people detect the true source of valence compared to when they closely overlap. Consistent our results, [16] (Experiment 3) observed stronger EC effects when stimuli were presented close together compared to when they were presented further away. Thus the current absolute distance findings are (at the mental level of analysis) in-line with an implicit attribution account (insofar as the moderating impact of distance on EC effects was due to an absolute distance difference in Experiments 1 and 3–5).

### Limitations and future directions

The current work is subject to several limitations and also opens up new directions in this research area. One obvious limitation was the ineffective impact of relative distance on EC effects. Despite our best efforts, the relative distance manipulations used here may simply have been too indirect to overcome the impact of mere contiguity on liking. Future work could increase the salience of such manipulations. For instance, imagine a scenario where two computer screens are linked together, and that in the distant condition the filler CS and US are on the opposite sides of the two screens, whereas in the close condition they are side-by-side. This may lead to even greater differences in focal stimulus evaluations than reported here. Second, it may be that people have to process distance *prior to* contiguity if the former is to overcome the latter’s impact on liking. Indeed, we found an impact of absolute and relative distance on explicit evaluations under such conditions (i.e., in Experiments 1 and 5) and no such impact when people could process contiguity before distance (as in Experiments 2–4). If anything, the processing of distance was secondary and optional in those latter experiments. Future work could carry out the aforementioned (multi-screen) study with the same task as used in Experiments 1 and 5.

The current work also focused on the spatial distance between stimuli. Others could examine if the symbolic meaning of pairings can be changed in other ways. For instance, one could examine if modifying the *temporal* distance between stimuli has an impact on liking (e.g., if EC effects are influenced by the timing or order of stimulus presentations). Previous work has attempted this and produced mixed findings in this regard. [23], [24] found some evidence that EC effects were stronger when stimuli were presented in a simultaneous (compared to sequential) manner whereas [16] found that individuals who processed the CS and US in close (compared to distant) temporal contiguity were more likely to show EC effects. Yet others have found no advantage for simultaneous over sequential presentations [25]. The same goes for stimulus ordering: both forward and backward conditioning often produce similar effects, [26]. Thus it may be that even here subtle attempts to shift EC effects are difficult to achieve once participants construe that a CS has been ‘paired’ with a US. Finally, future work could utilize alternative measures of implicit evaluations other than the IAT in order to test the robustness and generalizability of our findings at the implicit level.

## Conclusion

Our results lend support to the idea that EC effects can be moderated by manipulating one property of pairings (distance), but only when those manipulations are simple and direct (absolute distance) and less so when they are more complex and indirect (relative distance).
